# 2-(*tert*-Butoxy­carbonyl­amino)-2-(2-fluoro­phen­yl)acetic acid

**DOI:** 10.1107/S160053680904416X

**Published:** 2009-10-28

**Authors:** Muhammad Mahmood Anwar, Muhammad Saeed Iqbal, M. Nawaz Tahir

**Affiliations:** a Department of Chemistry, University of Sargodha, Sargodha, Pakistan; b Department of Chemistry, Government College University, Lahore, Pakistan; c Department of Physics, University of Sargodha, Sargodha, Pakistan

## Abstract

The title compound, C_13_H_16_FNO_4_, consists of conventional, centrosymmetric carboxyl­ate dimers. These dimers form infinite polymeric chains due to inter­molecular N—H⋯O hydrogen bonding. The 2-fluoro­phenyl unit is disordered over two sets of sites with an ocupancy ratio of 0.915 (3):0.085 (3).

## Related literature

For hydrogen-bond motifs, see: Bernstein *et al.* (1995 ▶). For a related structure, see: González-Cameno *et al.* (1996 ▶).
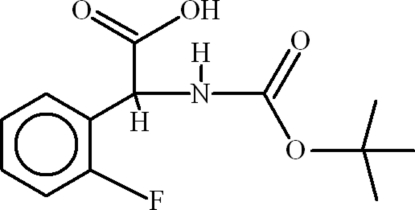


## Experimental

### 

#### Crystal data

C_13_H_16_FNO_4_
*M* *_r_* = 269.27
Triclinic, 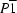

*a* = 5.3065 (3) Å
*b* = 10.6264 (6) Å
*c* = 12.4930 (6) Å
α = 106.175 (3)°
β = 95.175 (2)°
γ = 100.728 (3)°
*V* = 657.18 (6) Å^3^
*Z* = 2
Mo *K*α radiation
μ = 0.11 mm^−1^
*T* = 296 K
0.22 × 0.19 × 0.12 mm

#### Data collection

Bruker Kappa APEXII CCD diffractometer
Absorption correction: multi-scan (*SADABS*; Bruker, 2005 ▶) *T*_min_ = 0.974, *T*_max_ = 0.988
11718 measured reflections
2440 independent reflections
1826 reflections with *I* > 2σ(*I*)
*R*_int_ = 0.029

#### Refinement

*R*[*F* ^2^ > 2σ(*F* ^2^)] = 0.035
*wR*(*F* ^2^) = 0.094
*S* = 1.01
2440 reflections
198 parameters
H atoms treated by a mixture of independent and constrained refinement
Δρ_max_ = 0.14 e Å^−3^
Δρ_min_ = −0.15 e Å^−3^

### 

Data collection: *APEX2* (Bruker, 2007 ▶); cell refinement: *SAINT* (Bruker, 2007 ▶); data reduction: *SAINT*; program(s) used to solve structure: *SHELXS97* (Sheldrick, 2008 ▶); program(s) used to refine structure: *SHELXL97* (Sheldrick, 2008 ▶); molecular graphics: *ORTEP-3 for Windows* (Farrugia, 1997 ▶) and *PLATON* (Spek, 2009 ▶); software used to prepare material for publication: *WinGX* (Farrugia, 1999 ▶) and *PLATON*.

## Supplementary Material

Crystal structure: contains datablocks global, I. DOI: 10.1107/S160053680904416X/cs2125sup1.cif

Structure factors: contains datablocks I. DOI: 10.1107/S160053680904416X/cs2125Isup2.hkl

Additional supplementary materials:  crystallographic information; 3D view; checkCIF report

## Figures and Tables

**Table 1:** Hydrogen-bond geometry (Å, °)

*D*—H⋯*A*	*D*—H	H⋯*A*	*D*⋯*A*	*D*—H⋯*A*
N1—H1*A*⋯O3^i^	0.8600	2.3900	3.1883 (16)	155.00
O1—H1*O*⋯O2^ii^	0.8200	1.8200	2.6399 (16)	174.00

Symmetry codes: (i) 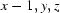; (ii) 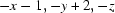.

**Fig. 1.**
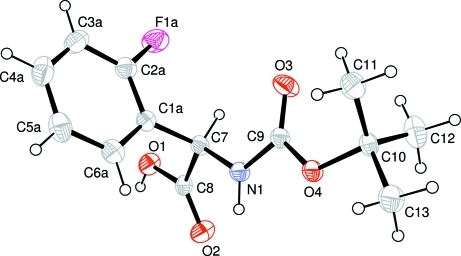
View of the title compound with the atom numbering scheme having atoms of greater occupancy ratio. The thermal ellipsoids are drawn at the 30% probability level. H-atoms are shown by small circles of arbitrary radii.

**Fig. 2.**
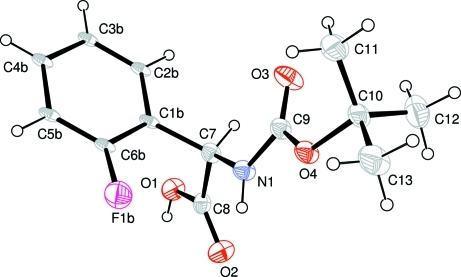
View of the title compound with the atom numbering scheme having atoms of smaller occupancy ratio. The thermal ellipsoids are drawn at the 30% probability level. H-atoms are shown by small circles of arbitrary radii.

**Fig. 3.**
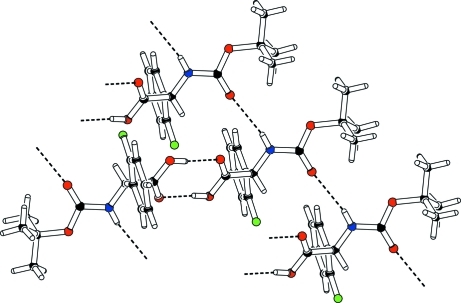
The partial packing (PLATON; Spek, 2009) which shows that molecules are dimerized and form polymeric chains.

## supplementary crystallographic information

### Comment

The cephalosporins are used as broad spectrum antibiotics. The title compound (I, Fig. 1) has been prepared for the synthesis of different fluoro substituted cephalosporins.

The crystal structure of (II) *N*-(t-Butoxycarbonyl)-2-phenylglycine (González-Cameno *et al.*, 1996) has been published. The title compound (I) differs from (II) due to substitution of F-atom on the benzene ring at *ortho* position.

In the molecules of the title compound 2-fluorophenyl moiety is disordered over two sets of sites with ocupancy ratio of 0.915 (3):0.085 (3). The dihedral angle between the disordered moiety is 7 (2)°. The molecules of the title compound form conventional dimers due to O–H···O type of intermolecular H-bondings with *R*_2_^2^(8) ring motifs (Bernstein *et al.*, 1995). The dimers are interlinked in the form of infinite one dimensional polymeric chains due to N—H···O type of intermolecular H-bonds (Table 1, Fig. 3). The benzene ring A (C1A—C6A), the group B (C7/C8/O1/O2) and C (N1/C9/O3/O4) are planar with r. m. s. deviations of 0.008, 0.0006 and 0.002 Å respectively, from the respective mean square planes. The major occupancy F1A-atom is at a distance of 0.0458 (74)Å from the plane of benzene ring. The dihedral angles between A/B, A/C and B/C are 80.63 (12), 80.14 (11) and 33.10 (8)°, respectively.

### Experimental

In first step 2-fluorophenyl glycine (0.169 g, 1 mmol) was dissolved in a solution of 1*M* NaHCO_3_ and cooled to 273 K. Then 2 equivalent of the di-*tert*-butyl dicarbonate (0.43 g, 2 mmol) was dissolved in 5 ml of 1,4 dioxane and also cooled to 273 K. Second solution was added dropwise to the former solution with constant stirring at 273 K for 2 h. Then the reaction mixture was stirred at ambient temperature for further 24 h. After this 25 ml of distilled water was added and aqous layer was extracted twice with ethyl acetate. The organic layer was back extracted with 1*M* NaHCO_3_ solution. The combined aqous layer was acidified to pH 2 with 10% HCl. The crude material was dissolved in ethyl acetate and evaporation of it affoarded the white prism of title compound (I).

### Refinement

The 2-fluorophenyl moiety is disordered. The benzene ring of the minor occupancy sites  was refined using AFIX 66 and EADP. The coordinates of H-atom attached with C7 were refined.

The H-atoms were positioned geometrically (O–H = 0.82 Å, N–H = 0.86 Å, C–H = 0.93–0.96 Å) and refined as riding with *U*_iso_(H) = 1.2*U*_eq_(carrier) or 1.5*U*_eq_(methyl C).

### Crystal data

**Table:**

C_13_H_16_FNO_4_	*Z* = 2
*M**_r_* = 269.27	*F*(000) = 284
Triclinic, *P*1	*D*_x_ = 1.361 Mg m^−^^3^
Hall symbol: -P 1	Mo *K*α radiation, λ = 0.71073 Å
*a* = 5.3065 (3) Å	Cell parameters from 2440 reflections
*b* = 10.6264 (6) Å	θ = 3.0–25.5°
*c* = 12.4930 (6) Å	µ = 0.11 mm^−^^1^
α = 106.175 (3)°	*T* = 296 K
β = 95.175 (2)°	Prism, white
γ = 100.728 (3)°	0.22 × 0.19 × 0.12 mm
*V* = 657.18 (6) Å^3^	

### Data collection

**Table:**

Bruker Kappa APEXII CCD diffractometer	2440 independent reflections
Radiation source: fine-focus sealed tube	1826 reflections with *I* > 2σ(*I*)
graphite	*R*_int_ = 0.029
Detector resolution: 7.70 pixels mm^-1^	θ_max_ = 25.5°, θ_min_ = 3.0°
ω scans	*h* = −6→6
Absorption correction: multi-scan (*SADABS*; Bruker, 2005)	*k* = −12→12
*T*_min_ = 0.974, *T*_max_ = 0.988	*l* = −15→15
11718 measured reflections	

### Refinement

**Table:**

Refinement on *F*^2^	Primary atom site location: structure-invariant direct methods
Least-squares matrix: full	Secondary atom site location: difference Fourier map
*R*[*F*^2^ > 2σ(*F*^2^)] = 0.035	Hydrogen site location: inferred from neighbouring sites
*wR*(*F*^2^) = 0.094	H atoms treated by a mixture of independent and constrained refinement
*S* = 1.01	*w* = 1/[σ^2^(*F*_o_^2^) + (0.0414*P*)^2^ + 0.1509*P*] where *P* = (*F*_o_^2^ + 2*F*_c_^2^)/3
2440 reflections	(Δ/σ)_max_ < 0.001
198 parameters	Δρ_max_ = 0.14 e Å^−^^3^
0 restraints	Δρ_min_ = −0.15 e Å^−^^3^

### Special details

**Table:**

Geometry. Bond distances, angles *etc*. have been calculated using the rounded fractional coordinates. All su's are estimated from the variances of the (full) variance-covariance matrix. The cell e.s.d.'s are taken into account in the estimation of distances, angles and torsion angles
Refinement. Refinement of *F*^2^ against ALL reflections. The weighted *R*-factor *wR* and goodness of fit *S* are based on *F*^2^, conventional *R*-factors *R* are based on *F*, with *F* set to zero for negative *F*^2^. The threshold expression of *F*^2^ > σ(*F*^2^) is used only for calculating *R*-factors(gt) *etc*. and is not relevant to the choice of reflections for refinement. *R*-factors based on *F*^2^ are statistically about twice as large as those based on *F*, and *R*- factors based on ALL data will be even larger.The coordinates of H7 were refined due to disorder in the adjacent ring and to check its role in H-bondings.

### Fractional atomic coordinates and isotropic or equivalent isotropic displacement parameters (Å^2^)

**Table:**

	*x*	*y*	*z*	*U*_iso_*/*U*_eq_	Occ. (<1)
F1A	0.3735 (2)	1.11138 (12)	0.22973 (11)	0.0633 (5)	0.915 (3)
O1	−0.1893 (2)	1.06206 (11)	0.08285 (10)	0.0471 (4)	
O2	−0.4647 (2)	0.86319 (11)	0.03740 (10)	0.0500 (4)	
O3	0.2330 (2)	0.73869 (12)	0.20577 (12)	0.0566 (5)	
O4	−0.1233 (2)	0.58870 (10)	0.21489 (10)	0.0423 (4)	
N1	−0.1695 (2)	0.77405 (12)	0.17674 (11)	0.0387 (4)	
C1A	−0.0132 (9)	1.0140 (3)	0.2816 (3)	0.0351 (8)	0.915 (3)
C2A	0.2103 (8)	1.1122 (5)	0.3090 (4)	0.0438 (9)	0.915 (3)
C3A	0.2789 (7)	1.2105 (5)	0.4113 (4)	0.0575 (11)	0.915 (3)
C4A	0.1121 (9)	1.2123 (4)	0.4889 (3)	0.0608 (13)	0.915 (3)
C5A	−0.1156 (8)	1.1190 (3)	0.4644 (3)	0.0565 (10)	0.915 (3)
C6A	−0.1775 (8)	1.0200 (3)	0.3618 (3)	0.0456 (9)	0.915 (3)
C7	−0.0754 (3)	0.90636 (15)	0.16792 (13)	0.0350 (5)	
C8	−0.2649 (3)	0.94137 (15)	0.08892 (12)	0.0345 (5)	
C9	0.0012 (3)	0.70217 (15)	0.19954 (13)	0.0363 (5)	
C10	0.0182 (3)	0.48439 (15)	0.22791 (14)	0.0419 (5)	
C11	0.2318 (4)	0.54035 (19)	0.32723 (16)	0.0569 (7)	
C12	0.1142 (4)	0.4254 (2)	0.11932 (17)	0.0687 (8)	
C13	−0.1926 (4)	0.38239 (18)	0.2507 (2)	0.0670 (8)	
C6B	−0.167 (6)	1.049 (3)	0.355 (3)	0.024 (4)	0.085 (3)
F1B	−0.3871 (8)	0.9486 (3)	0.3406 (3)	0.065 (5)	0.085 (3)
C1B	0.004 (8)	1.032 (4)	0.276 (3)	0.024 (4)	0.085 (3)
C2B	0.249 (7)	1.117 (4)	0.300 (4)	0.024 (4)	0.085 (3)
C3B	0.323 (5)	1.219 (4)	0.402 (4)	0.024 (4)	0.085 (3)
C4B	0.152 (6)	1.236 (3)	0.480 (3)	0.024 (4)	0.085 (3)
C5B	−0.093 (5)	1.151 (3)	0.457 (3)	0.024 (4)	0.085 (3)
H1A	−0.33354	0.74109	0.16751	0.0464*	
H11B	0.36743	0.60249	0.31082	0.0854*	
H11C	0.30010	0.46833	0.34204	0.0854*	
H12A	0.24638	0.49209	0.10625	0.1030*	
H12B	−0.02735	0.39635	0.05819	0.1030*	
H12C	0.18483	0.34993	0.12422	0.1030*	
H13A	−0.33342	0.35205	0.18919	0.1004*	
H13B	−0.25423	0.42301	0.31914	0.1004*	
H13C	−0.12400	0.30732	0.25826	0.1004*	
H1O	−0.30395	1.08012	0.04505	0.0565*	
H3A	0.43443	1.27405	0.42737	0.0689*	0.915 (3)
H4A	0.15449	1.27770	0.55882	0.0727*	0.915 (3)
H5A	−0.22955	1.12194	0.51697	0.0677*	0.915 (3)
H6A	−0.33266	0.95622	0.34619	0.0547*	0.915 (3)
H7	0.083 (3)	0.9064 (16)	0.1350 (13)	0.0419*	
H11A	0.16404	0.58594	0.39224	0.0854*	
H2B	0.36308	1.10485	0.24762	0.0290*	0.085 (3)
H3B	0.48669	1.27522	0.41761	0.0290*	0.085 (3)
H4B	0.20175	1.30421	0.54823	0.0290*	0.085 (3)
H5B	−0.20681	1.16284	0.50885	0.0290*	0.085 (3)

### Atomic displacement parameters (Å^2^)

**Table:**

	*U*^11^	*U*^22^	*U*^33^	*U*^12^	*U*^13^	*U*^23^
F1A	0.0511 (8)	0.0626 (8)	0.0711 (9)	−0.0036 (6)	0.0154 (6)	0.0206 (6)
O1	0.0543 (7)	0.0359 (6)	0.0548 (7)	0.0109 (5)	−0.0032 (6)	0.0225 (5)
O2	0.0532 (8)	0.0414 (7)	0.0550 (7)	0.0034 (6)	−0.0086 (6)	0.0238 (6)
O3	0.0364 (7)	0.0523 (7)	0.0964 (10)	0.0152 (6)	0.0153 (6)	0.0418 (7)
O4	0.0365 (6)	0.0306 (6)	0.0652 (8)	0.0112 (5)	0.0039 (5)	0.0216 (5)
N1	0.0339 (7)	0.0310 (7)	0.0553 (8)	0.0096 (6)	0.0024 (6)	0.0195 (6)
C1A	0.0404 (14)	0.0314 (14)	0.0393 (11)	0.0130 (11)	0.0010 (9)	0.0182 (10)
C2A	0.044 (2)	0.0424 (13)	0.0483 (15)	0.0121 (15)	0.0053 (12)	0.0176 (11)
C3A	0.056 (2)	0.0427 (14)	0.064 (2)	0.0053 (15)	−0.0120 (19)	0.0108 (13)
C4A	0.082 (3)	0.053 (2)	0.0453 (14)	0.0285 (18)	−0.0037 (16)	0.0066 (15)
C5A	0.0739 (19)	0.061 (2)	0.0448 (14)	0.0320 (17)	0.0151 (12)	0.0192 (15)
C6A	0.0497 (14)	0.0474 (19)	0.0475 (12)	0.0162 (13)	0.0101 (11)	0.0224 (13)
C7	0.0363 (8)	0.0308 (8)	0.0424 (9)	0.0108 (7)	0.0073 (7)	0.0157 (7)
C8	0.0425 (9)	0.0306 (8)	0.0333 (8)	0.0116 (7)	0.0072 (7)	0.0115 (7)
C9	0.0377 (9)	0.0329 (8)	0.0420 (9)	0.0116 (7)	0.0056 (7)	0.0149 (7)
C10	0.0418 (9)	0.0309 (8)	0.0578 (10)	0.0166 (7)	0.0037 (8)	0.0169 (8)
C11	0.0573 (11)	0.0504 (11)	0.0674 (12)	0.0206 (9)	−0.0027 (9)	0.0227 (9)
C12	0.0867 (15)	0.0594 (12)	0.0668 (13)	0.0373 (11)	0.0143 (11)	0.0153 (10)
C13	0.0565 (12)	0.0412 (10)	0.1141 (18)	0.0152 (9)	0.0098 (11)	0.0390 (11)
C6B	0.012 (6)	0.022 (6)	0.047 (8)	0.004 (5)	0.007 (5)	0.022 (5)
F1B	0.053 (9)	0.062 (9)	0.075 (9)	0.004 (7)	0.023 (7)	0.015 (7)
C1B	0.012 (6)	0.022 (6)	0.047 (8)	0.004 (5)	0.007 (5)	0.022 (5)
C2B	0.012 (6)	0.022 (6)	0.047 (8)	0.004 (5)	0.007 (5)	0.022 (5)
C3B	0.012 (6)	0.022 (6)	0.047 (8)	0.004 (5)	0.007 (5)	0.022 (5)
C4B	0.012 (6)	0.022 (6)	0.047 (8)	0.004 (5)	0.007 (5)	0.022 (5)
C5B	0.012 (6)	0.022 (6)	0.047 (8)	0.004 (5)	0.007 (5)	0.022 (5)

### Geometric parameters (Å, °)

**Table:**

F1A—C2A	1.372 (5)	C5B—C6B	1.39 (5)
F1B—C6B	1.39 (3)	C7—C8	1.513 (2)
O1—C8	1.296 (2)	C10—C11	1.504 (3)
O2—C8	1.216 (2)	C10—C12	1.504 (3)
O3—C9	1.207 (2)	C10—C13	1.512 (3)
O4—C10	1.486 (2)	C2B—H2B	0.9300
O4—C9	1.335 (2)	C3A—H3A	0.9300
O1—H1O	0.8200	C3B—H3B	0.9300
N1—C7	1.440 (2)	C4A—H4A	0.9300
N1—C9	1.348 (2)	C4B—H4B	0.9300
N1—H1A	0.8600	C5A—H5A	0.9300
C1A—C2A	1.371 (6)	C5B—H5B	0.9200
C1A—C6A	1.382 (6)	C6A—H6A	0.9300
C1A—C7	1.518 (4)	C7—H7	0.969 (16)
C1B—C2B	1.39 (6)	C11—H11A	0.9600
C1B—C7	1.57 (4)	C11—H11B	0.9600
C1B—C6B	1.40 (5)	C11—H11C	0.9600
C2A—C3A	1.374 (7)	C12—H12A	0.9600
C2B—C3B	1.39 (7)	C12—H12B	0.9600
C3A—C4A	1.369 (6)	C12—H12C	0.9600
C3B—C4B	1.39 (5)	C13—H13B	0.9600
C4A—C5A	1.362 (6)	C13—H13C	0.9600
C4B—C5B	1.39 (4)	C13—H13A	0.9600
C5A—C6A	1.379 (5)		
			
F1A···O1^i^	3.1159 (16)	C12···O3	3.121 (3)
F1A···C6A^i^	3.223 (4)	C13···F1A^viii^	3.266 (2)
F1A···C8^i^	3.189 (2)	C3A···H11C^vi^	3.0800
F1A···C13^ii^	3.266 (2)	C3B···H11C^vi^	2.9700
F1B···N1	2.841 (4)	C3B···H5B^i^	2.9600
F1B···C8	3.250 (4)	C4A···H11A^vii^	2.9600
F1B···O3^iii^	2.738 (4)	C4B···H11A^vii^	2.9900
F1B···C2B^iii^	2.97 (4)	C5B···H11A^vii^	3.0200
F1A···H7	2.347 (17)	C5B···H3B^iii^	2.8700
F1B···H2B^iii^	2.7300	C6B···H2B^iii^	2.9600
F1B···H1A	2.7000	C8···H1O^v^	2.6400
O1···C8^iv^	3.3671 (19)	C9···H12A	2.8200
O1···O1^iv^	3.2057 (16)	C9···H11B	2.8200
O1···O2^v^	2.6399 (16)	H1A···O3^iii^	2.3900
O1···F1A^iii^	3.1159 (16)	H1A···O2	2.4700
O1···C2A	3.228 (5)	H1A···F1B	2.7000
O1···C2B	3.25 (4)	H1O···O1^v^	2.9000
O2···C8^v^	3.375 (2)	H1O···O2^v^	1.8200
O2···O3^iii^	3.1807 (18)	H1O···H1O^v^	2.3800
O2···N1	2.7130 (17)	H1O···C8^v^	2.6400
O2···O1^v^	2.6399 (16)	H2B···F1B^i^	2.7300
O3···C11	2.920 (2)	H2B···H7	2.3300
O3···F1B^i^	2.738 (4)	H2B···C6B^i^	2.9600
O3···C12	3.121 (3)	H3B···H13B^ii^	2.5400
O3···C1A	3.356 (4)	H3B···C5B^i^	2.8700
O3···O2^i^	3.1807 (18)	H3B···H5B^i^	2.5500
O3···N1^i^	3.1883 (16)	H4A···O4^vii^	2.8200
O1···H1O^v^	2.9000	H4A···H11A^vii^	2.4400
O1···H13C^vi^	2.8400	H4B···H11A^vii^	2.5100
O2···H1O^v^	1.8200	H5B···C3B^iii^	2.9600
O2···H7^iii^	2.843 (16)	H5B···H3B^iii^	2.5500
O2···H1A	2.4700	H5B···H11A^vii^	2.5700
O3···H7	2.423 (17)	H6A···N1	2.7700
O3···H1A^i^	2.3900	H7···O2^i^	2.843 (16)
O3···H11B	2.3700	H7···F1A	2.347 (17)
O3···H12A	2.5900	H7···O3	2.423 (17)
O4···H4A^vii^	2.8200	H7···H2B	2.3300
N1···O3^iii^	3.1883 (16)	H11A···C4A^vii^	2.9600
N1···O2	2.7130 (17)	H11A···H13B	2.4700
N1···F1B	2.841 (4)	H11A···C5B^vii^	3.0200
N1···H6A	2.7700	H11A···H4A^vii^	2.4400
C1A···O3	3.356 (4)	H11A···C4B^vii^	2.9900
C2A···O1	3.228 (5)	H11A···H4B^vii^	2.5100
C2B···O1	3.25 (4)	H11A···H5B^vii^	2.5700
C2B···F1B^i^	2.97 (4)	H11B···H12A	2.4600
C2B···C6B^i^	3.37 (5)	H11B···O3	2.3700
C3A···C5A^i^	3.584 (6)	H11B···C9	2.8200
C3B···C6B^i^	3.53 (5)	H11C···H13C	2.5000
C3B···C5B^i^	3.37 (4)	H11C···C3A^ix^	3.0800
C4A···C6A^vii^	3.530 (5)	H11C···H13B^i^	2.5300
C5A···C5A^vii^	3.302 (5)	H11C···C3B^ix^	2.9700
C5A···C6A^vii^	3.369 (5)	H12A···H11B	2.4600
C5A···C3A^iii^	3.584 (6)	H12A···O3	2.5900
C5B···C3B^iii^	3.37 (4)	H12A···C9	2.8200
C6A···C5A^vii^	3.369 (5)	H12B···H13A	2.4800
C6A···C4A^vii^	3.530 (5)	H12C···H13C	2.5200
C6A···F1A^iii^	3.223 (4)	H13A···H12B	2.4800
C6B···C3B^iii^	3.53 (5)	H13B···H3B^viii^	2.5400
C6B···C2B^iii^	3.37 (5)	H13B···H11A	2.4700
C8···O2^v^	3.375 (2)	H13B···H11C^iii^	2.5300
C8···O1^iv^	3.3671 (19)	H13C···H12C	2.5200
C8···F1A^iii^	3.189 (2)	H13C···O1^ix^	2.8400
C8···F1B	3.250 (4)	H13C···H11C	2.5000
C11···O3	2.920 (2)		
			
C9—O4—C10	120.76 (12)	O4—C10—C13	101.89 (13)
C8—O1—H1O	109.00	C11—C10—C12	112.88 (15)
C7—N1—C9	119.57 (12)	C3B—C2B—H2B	120.00
C9—N1—H1A	120.00	C1B—C2B—H2B	120.00
C7—N1—H1A	120.00	C2A—C3A—H3A	121.00
C6A—C1A—C7	122.5 (3)	C4A—C3A—H3A	121.00
C2A—C1A—C6A	116.5 (4)	C2B—C3B—H3B	120.00
C2A—C1A—C7	121.0 (4)	C4B—C3B—H3B	120.00
C6B—C1B—C7	118 (3)	C3A—C4A—H4A	120.00
C2B—C1B—C7	122 (3)	C5A—C4A—H4A	120.00
C2B—C1B—C6B	120 (4)	C3B—C4B—H4B	120.00
F1A—C2A—C1A	117.9 (4)	C5B—C4B—H4B	120.00
C1A—C2A—C3A	123.5 (4)	C4A—C5A—H5A	120.00
F1A—C2A—C3A	118.6 (4)	C6A—C5A—H5A	120.00
C1B—C2B—C3B	120 (4)	C4B—C5B—H5B	120.00
C2A—C3A—C4A	118.2 (4)	C6B—C5B—H5B	120.00
C2B—C3B—C4B	120 (3)	C5A—C6A—H6A	119.00
C3A—C4A—C5A	120.5 (4)	C1A—C6A—H6A	119.00
C3B—C4B—C5B	120 (3)	C1A—C7—H7	107.8 (10)
C4A—C5A—C6A	120.1 (4)	C8—C7—H7	106.5 (10)
C4B—C5B—C6B	120 (3)	C1B—C7—H7	103.1 (18)
C1A—C6A—C5A	121.2 (4)	N1—C7—H7	108.1 (10)
C1B—C6B—C5B	120 (3)	C10—C11—H11B	109.00
F1B—C6B—C1B	119 (3)	C10—C11—H11C	109.00
F1B—C6B—C5B	120 (3)	C10—C11—H11A	109.00
N1—C7—C8	111.29 (13)	H11A—C11—H11B	109.00
N1—C7—C1A	112.66 (17)	H11A—C11—H11C	109.00
C1A—C7—C8	110.2 (2)	H11B—C11—H11C	109.00
C1B—C7—C8	105.9 (15)	C10—C12—H12A	110.00
N1—C7—C1B	120.9 (15)	C10—C12—H12B	109.00
O2—C8—C7	122.76 (15)	C10—C12—H12C	109.00
O1—C8—O2	124.74 (15)	H12A—C12—H12B	110.00
O1—C8—C7	112.50 (13)	H12B—C12—H12C	109.00
O3—C9—O4	126.25 (15)	H12A—C12—H12C	109.00
O4—C9—N1	110.30 (13)	C10—C13—H13B	109.00
O3—C9—N1	123.45 (15)	C10—C13—H13C	109.00
C12—C10—C13	110.98 (16)	C10—C13—H13A	109.00
C11—C10—C13	110.15 (16)	H13A—C13—H13C	109.00
O4—C10—C11	110.98 (14)	H13B—C13—H13C	109.00
O4—C10—C12	109.43 (14)	H13A—C13—H13B	109.00
			
C10—O4—C9—N1	172.81 (13)	C2A—C1A—C7—N1	135.7 (3)
C9—O4—C10—C12	−66.19 (19)	C6A—C1A—C2A—F1A	−178.0 (3)
C9—O4—C10—C13	176.28 (15)	C2A—C1A—C7—C8	−99.4 (4)
C9—O4—C10—C11	59.04 (19)	C6A—C1A—C7—C8	79.6 (4)
C10—O4—C9—O3	−7.9 (2)	C6A—C1A—C7—N1	−45.3 (4)
C9—N1—C7—C8	151.29 (14)	F1A—C2A—C3A—C4A	178.6 (4)
C7—N1—C9—O4	174.06 (13)	C1A—C2A—C3A—C4A	−1.6 (7)
C9—N1—C7—C1A	−84.4 (3)	C2A—C3A—C4A—C5A	−0.2 (7)
C7—N1—C9—O3	−5.2 (2)	C3A—C4A—C5A—C6A	1.4 (6)
C7—C1A—C2A—F1A	1.1 (6)	C4A—C5A—C6A—C1A	−0.8 (6)
C7—C1A—C2A—C3A	−178.7 (4)	N1—C7—C8—O1	177.52 (12)
C2A—C1A—C6A—C5A	−0.9 (6)	N1—C7—C8—O2	−2.7 (2)
C7—C1A—C6A—C5A	180.0 (3)	C1A—C7—C8—O1	51.8 (2)
C6A—C1A—C2A—C3A	2.2 (7)	C1A—C7—C8—O2	−128.4 (2)

Symmetry codes: (i) *x*+1, *y*, *z*; (ii) *x*+1, *y*+1, *z*; (iii) *x*−1, *y*, *z*; (iv) −*x*, −*y*+2, −*z*; (v) −*x*−1, −*y*+2, −*z*; (vi) *x*, *y*+1, *z*; (vii) −*x*, −*y*+2, −*z*+1; (viii) *x*−1, *y*−1, *z*; (ix) *x*, *y*−1, *z*.

### Hydrogen-bond geometry (Å, °)

**Table:**

*D*—H···*A*	*D*—H	H···*A*	*D*···*A*	*D*—H···*A*
N1—H1A···O3^iii^	0.8600	2.3900	3.1883 (16)	155.00
O1—H1O···O2^v^	0.8200	1.8200	2.6399 (16)	174.00

Symmetry codes: (iii) *x*−1, *y*, *z*; (v) −*x*−1, −*y*+2, −*z*.
